# A Qualitative Study on Gendered Barriers to Livestock Vaccine Uptake in Kenya and Uganda and Their Implications on Rift Valley Fever Control

**DOI:** 10.3390/vaccines7030086

**Published:** 2019-08-08

**Authors:** Edna Mutua, Nicoline de Haan, Dan Tumusiime, Christine Jost, Bernard Bett

**Affiliations:** 1 International Livestock Research Institute, Post Office Box 30709, Nairobi 00100, Kenya; 2 Ministry of Agriculture, Animal Industry and Fisheries, Post Office Box 102, Plot 16-18 Lugard Avenue, Entebbe, Uganda; 3 Global Health Support Initiative III, Social Solutions International, United States Agency for International Development Office of US Foreign Disaster Assistance, 1300 Pennsylvania Ave North West, Washington, DC 20523, USA

**Keywords:** livestock, vaccines, rift valley fever, gender, barriers, uptake, Uganda, Kenya

## Abstract

Rift Valley fever (RVF) is a zoonotic disease of great public health and economic importance transmitted by mosquitoes. The main method of preventing the disease is vaccination of susceptible livestock before outbreaks occur. Studies on RVF vaccines have focused on the production processes, safety, and efficacy standards but those on uptake and adoption levels are rare. This study sought to understand the barriers faced by men and women farmers in the uptake of livestock vaccines to inform strategies for optimizing the use of vaccines against RVF in East Africa. The cross-sectional qualitative study utilized the pairwise ranking technique in sex disaggregated focus group discussions to identify and rank these barriers. Results indicate that men and women farmers experience barriers to vaccine uptake differentially. The barriers include the direct and indirect cost of vaccines, distances to vaccination points, availability of vaccination crushes, intra-household decision making processes and availability of information on vaccination campaigns. The study concludes that vaccine provision does not guarantee uptake at the community level. Hence, these barriers should be considered while designing vaccination strategies to enhance community uptake because vaccine uptake is a complex process which requires buy-in from men and women farmers, veterinary departments, county/district and national governments, and vaccine producers.

## 1. Introduction

Rift Valley fever (RVF) is a viral zoonotic disease of great public health and economic significance. The disease is caused by the Rift Valley fever virus (RVFv), a *Phlebovirus* of the *Phenuiviridae* family [1], which is transmitted by multiple mosquito species [2,3,4]. RVF is a climate sensitive disease whose occurrence in East Africa has been associated with above normal rains and flooding [2,5], which establish ample breeding grounds for its vectors [2,6]. Cattle, sheep, goats, and camels are highly susceptible to the virus. Unlike in livestock where RVF disease transmission is by mosquitoes [7], the vectors play a role in disease spread in humans by causing mild infections whereas most severe infections are through contact with infected animal tissues and secretions, such as meat and blood, respectively [2,4,8]. RVF is listed as a notifiable disease in Kenya [9] and has been reported to occur 12 times, with the latest documented outbreak occurring in 2018 [10,11]. It has been estimated that during the 2006–2007 outbreak in Kenya—the most severe outbreak recorded—the country incurred losses of US $32 million from animal deaths, loss of productivity, trade bans imposed on livestock and livestock products, and disruption of related services [12]. In Uganda, RVF disease outbreak was also last reported in 2018 [13]. Before then, there have been three reported outbreaks in 1968, 2016, and 2017 [13,14]. Currently, there are no estimates of the economic impacts of any of the outbreaks that have occurred in Uganda.

Due to the possible debilitating impacts of RVF outbreaks, multiple interventions are often used singly or in combination to prevent and control the disease. These include vector control; quarantines; bans on slaughter and trade of livestock and livestock products; information dissemination on avoiding risky handling of infected animals and livestock products; and livestock vaccination. Vector control often involves spraying insecticides in mosquito breeding and preferred resting places, thus disrupting the disease’s transmission cycle [6]. Livestock quarantines limit the potential for RVF spread through movement of infected animals from endemic to non-endemic areas as well as dissemination of the virus and contact between infected and susceptible animals [15]. Infected vectors can also, through their movement, introduce the virus to uninfected areas [15]. Although difficult to enforce, bans on slaughter and sale of livestock products and avoidance of risky handling of infected livestock and livestock products in outbreak periods can be very effective in reducing human exposure [2,16,17,18,19].

Vaccination of susceptible livestock against RVF is the most effective prevention and control measure for RVF. It can be used reliably to prevent epidemics if it is conducted early enough to allow animals to acquire desired immunity status before an outbreak occurs [20,21]. Livestock vaccination not only reduces the risk of infection in animals but it also minimizes the risk of zoonotic transmissions. Some of the vaccines that have been developed for RVF control in livestock include the Smithburn vaccine, Clone 13, and MP12 [5]. Of these, only the Smithburn vaccine is produced commercially and used in East Africa. However, the vaccine is thought to be expensive for a majority of producers and users as it is produced from a live attenuated virus [5]. The vaccine is also not recommended for use in young and gestating animals because it may cause disease and abortions, respectively [5]. Despite these shortcomings, only the Smithburn vaccine is approved for RVF prevention in Kenya. Until 2016, Uganda was categorized as having a low risk of RVF and hence livestock vaccination had neither been adopted as part of its control measures nor was the disease listed as state controlled [13]. Consequently, RVF vaccination has not been approved in the country and no guidelines on how vaccination can be done are available [13].

Studies on vaccines, RVF vaccines included, have primarily focused on the vaccine production processes, safety and efficacy standards [5,15,22,23,24,25,26,27,28,29]. Thus, very few studies have been conducted on vaccine uptake (in this study defined as the process farmers take from when they receive livestock vaccination information to consenting to have their animals vaccinated and presenting their animals for vaccination) and adoption (defined as continuous use when needed even without the intervention of veterinary departments). The few that have been conducted have also tended not to pay attention to gender in the analysis, presenting the experiences of farmers as a homogeneous group. This study determined gendered barriers faced by men and women farmers in the utilization of vaccines provided by veterinary departments at a community level. The study focused on experiences from two sites in Kenya where the Smithburn vaccine had been provided to community members and two sites in Uganda where vaccination against RVF had never been conducted. The four sites selected for the study had experienced RVF outbreaks in the recent past. The study established that farmers had little or no knowledge on RVF in the four study sites, including Murang’a and Kwale, where the vaccine had been provided at no cost to the farmer. Thus, the study made reference to the uptake of other vaccines to understand how prevailing practice and perceptions can affect RVF vaccine uptake.

## 2. Materials and Methods

### 2.1. Study Area

The study was implemented in Murang’a and Kwale Counties in Kenya and Ibanda and Arua Districts in Uganda. The study sites in Kenya and Uganda were selected purposively; those selected in Kenya had vaccinated their livestock against RVF in the recent past while those in Uganda had recent RVF outbreaks but no vaccination against the disease was done. Farmers in Murang’a, Kenya, practiced dairy farming in a zero-grazing system, while those in Kwale were either agro-pastoral or pastoral. In Uganda, the farmers were primarily agro-pastoral.

### 2.2. Study Design, Sampling and Data Collection

The study applied a cross-sectional research design. Focus Group Discussions (FGDs) that comprised of 8–12 discussants were used to collect data. In Kenya, a total of 30 FGDs were conducted, 14 in Murang’a and 16 in Kwale, while in Uganda, 28 FGDs were conducted,14 in each site. In all, 58 FGDs were conducted in this study. In each country, half of the FGDs were men only groups and the other half women only. All focus group discussants were selected purposively to enable the researchers to engage participants that met the inclusion criteria for the study. To participate in FGDs, participants had to be current livestock keepers from areas that had received the RVF vaccines in Kenya or recently suffered an RVF outbreak in Uganda. All focus group discussion proceedings were tape-recorded, and notes were also taken during the discussions.

In all FGDs, pairwise ranking exercises were conducted to identify barriers with the greatest impact on livestock vaccine uptake for men and women farmers as outlined in Gay et al., 2016 [30]. In each FGD, participants first listed barriers that they faced, then explained the reasons why the barriers were considered so. Thereafter, each of the barriers were compared in pairs for impact and the outcome was documented in a matrix. After all possible comparisons were completed, the outcomes were tallied and ranked, with the highest score attaining the highest rank. Data were collected in Swahili in Murang’a and Kwale Counties in Kenya; while in Uganda, Lugbara was used in the Arua District and Runyankole/Rukiga in the Ibanda District. Prior to conducting the data collection, the FGD guide was pretested in Murang’a, Ibanda, and Arua to test the suitability of questions and the ability of participants to comprehend, and relevant adjustments were made. A key outcome of the pretest was ensuring that the terms used for vaccines were clear to farmers and there was no confusion with other injectable veterinary medicines. The pretest sites were excluded from the study.

### 2.3. Data Management and Analysis

All audio data were translated and transcribed into English by the data collection team, which had a good command of English and Swahili, Lugbara, or Runyankole/Rukiga. The transcripts were verified by comparing the audio files and scripts with the field notes taken during the discussions. Data were managed and coded into emergent themes in Nvivo 12 (QSR International, Melbourne, Victoria, Australia) and analyzed inductively using the content analysis method.

### 2.4. Ethics

The study received ethical clearance from the International Livestock Research Institute’s Research and Ethics Committee referenced ILRI-IREC2017-19. Prior to taking part in the study, all participants were provided with details about the study topic; the methods that would be used in data collection; the estimated time the discussions would take; use of recorders and note-taking; and how the data collected will be managed and used. Participants were given the opportunity to ask questions and receive adequate answers and thereafter consented voluntarily by signing a consent form or if illiterate, providing a thumb print in place of a signature. All focus group discussants were aged eighteen years and above, which is Kenya’s and Uganda’s consenting age.

## 3. Results

Study results are organized into three sub-sections. The first, Section 3.1., focuses on the demographic characteristics of the study participants. The second, Section 3.2., presents the barriers to vaccine uptake listed by men (M) and women (W) farmers. The last, Section 3.3., focuses on the ranking of key barriers to vaccine uptake for men and women farmers based on self-assessment and triangulation with pairwise ranking exercises.

### 3.1. Demographic Characteristics

A total of 645 discussants, 322 men and 323 women, participated in 58 FGDs comprising of 14 FGDs each in Murang’a, Ibanda, and Arua and 16 in Kwale (Table 1). Overall, the majority of the participants had primary education; 53% in Murang’a, 51.5% in Kwale, 59.6% in Ibanda, and 59.6% in Arua. However, it was only in Murang’a and Arua where more women discussants had primary education compared to men. In Murang’a, Kwale, and Arua, more men had secondary education compared to women. Except in Kwale, more men discussants had tertiary education. The highest number of discussants without formal education was recorded in Kwale and Ibanda among women. Discussants ages ranged from 20–82 years, with a mean age of 45.5 years and a median age of 45 years. Cumulatively, men discussants were older than women. By site, Murang’a had the oldest discussants, while Arua had the youngest. Among the livestock species susceptible to RVF, the farmers kept cattle, sheep, and goats. However, the farmers preferred presenting cattle for vaccination compared to goats and sheep.

### 3.2. Barriers to Vaccine Uptake Encountered by Men and Women Farmers

Men farmers in Murang’a identified 16 barriers to vaccine uptake, Kwale 17, Ibanda 15, and Arua 22, while women identified 15, 20, 14, and 16, respectively. By site, combining both men’s and women’s contributions, a total of 18 vaccine uptake barriers were identified in Murang’a, 23 in Kwale, 21 in Ibanda and 29 in Arua. Of the identified barriers, both men and women identified 13 similar barriers in Murang’a, 14 in Kwale, 8 in Ibanda, and 9 in Arua. Table 2 provides a list of barriers faced by men and women farmers. Site-specific barrier lists are provided as part of the supplementary materials (Table S1–Table S4). The following are the main barriers identified in the four study sites:

(a) Vaccine costs:

Three vaccine pricing structures were used in the four case studies, each resulting in different vaccine costs to the farmer. In Murang’a, farmers were required to pay a cost of 50 Kenya shillings (0.5 United States Dollar (USD)) per animal if they took their animals for vaccination in a central place and 100 Kenya shillings (1 USD) if they preferred vaccinations to be done at home since it was not standard practice. Where farmers needed to hire additional labor to move cattle to vaccination points, extra costs were incurred. Where a farmer could not afford the vaccination fees for all eligible animals, preference was given to the more productive and valued animals and the rest were left out. In all the 14 FGDs, farmers reported the vaccine cost as high as not all community members could comfortably afford, particularly if they had many animals.

In Kwale, the County government bore the cost of the vaccine and delivery. However, households that did not participate in building community crushes or intended to use community managed cattle dips as vaccination venues were charged a cost of 5–10 Kenya shillings (0.05 USD–0.1USD) for every vaccinated head of cattle or goat by cattle dip/crush committees (7 FGDs; 3-M, 4-W). The fee was levied by other community members and dip committees to aid in the maintenance of the facilities. The fee was considered a deterrent especially by farmers with large livestock numbers.

Except for one FGD, all other discussion groups in Ibanda (14FGDs; 7-M, 7-W) and Arua (13 FGDs, 7-M, 6-W) cited cost as a major barrier to vaccine uptake. In these areas, farmers were required to pay for the indirect costs incurred by the veterinary departments as they delivered livestock vaccines, but not the direct cost of the vaccine. The prices ranged from 2000 Uganda shillings (0.5 USD) per head of cattle to 3000 Uganda shillings (0.78 USD) in some circumstances. The perception of the vaccine cost being high was also driven by the belief that farmers should not have to pay for government provided vaccines. Where farmers could not afford to have all their eligible animals vaccinated, they either stayed away from the exercise or gave preference to the more productive animals and left out unproductive ones.

(b) Choice of and distances to vaccination points:

Farmers in all 14 FGDs conducted in Murang’a identified the choice of livestock vaccination points as a vaccine uptake challenge. The farmers kept zero grazed animals, which were unaccustomed to long-range movements. Consequently, moving the animals to vaccination points was difficult. This was further compounded by the distance that had to be covered and the number of animals that a farmer wanted to be vaccinated. Farmers living in distant places from the vaccination points experienced more difficulties moving their animals than those living closer. Moving more than one animal to vaccination points was more challenging. Difficulty with moving animals to vaccination points introduced an indirect cost of vaccines since farmers who were not able to manage their animals hired labor to assist with the task. Similarly, in Kwale, farmers from all 16 FGDs identified the long distances to livestock vaccination points as a barrier to livestock vaccine uptake. Although most farmers in the area kept local livestock breeds that were accustomed to movement in search of water and pasture, distances to vaccination points were amplified by the limited number of areas with crushes where livestock vaccination was conducted as reported in 14 FGDs (7-M, 7-W). According to six FGDs (3-M, 3-W), farmers in Arua were also faced with having to trek lengthy distances to access vaccination services based on proximity to selected vaccination points.

(c) Fear of disease spread during livestock vaccination campaigns:

At the vaccination points, farmers cited the possibility of their animals being infected with disease from the use of one needle to vaccinate many animals and through contact between animals from many households as barriers to vaccine uptake. Farmers from 13 FGDs (7-M, 6-W) in Murang’a and 3 FGDs (2-M, 1-W) in Ibanda were concerned that their animals could be infected with diseases from other animals through the use of a single needle to vaccinate many animals. This thinking was drawn from practice in human health where needle sharing is not acceptable due to the risk of spreading disease from one person to another. In Murang’a 11 FGDs (7-M, 4-W) and Kwale 9 FGDs (4-M, 5-W), reported the possibility of disease spread through contact of animals from different households and indirectly through the spread of pests such as ticks and tsetse flies as a barrier.

(d) Fear of vaccine side effects:

Upon vaccination, farmers from 11 FGDs (6-M, 5-W) in Murang’a were concerned about the possible side effects of the administered vaccine. The vaccine side effects identified included abortions among in-calf animals (9 FGDs; 5-M, 4-W), decrease in milk production for few days from the day of vaccination (3 FGDs;1-M, 2-W), animals swelling on the vaccination site (2 FGDs; 2-W) and becoming sickly (1 FGD; W). Farmers tended not to present in-calf animals for vaccination to prevent the occurrence of abortions, save the calf, and safeguard their investment in artificial insemination services used to fertilize the animal. In Kwale, three FGDs (1-M, 2-W) identified tail tip loss (2 FGDs; 1-M, 1-W) and abortions (1 FGD; W) as the vaccine side effects they had encountered. In Ibanda, 11 FGDs (5-M, 6-W), identified vaccine side effects such as reduced milk yields (1 FGD; W), abortions in in-calf animals (6 FGDs; 3-M, 3-W), animals becoming sick or dying (6 FGDs; 3-M, 3-W), and tail tips falling off after vaccination (1 FGD; M) as deterrents to vaccine uptake. Of the identified vaccine side effects, abortions were the main concern for farmers from the three sites.

(e) Disinterest in vaccines:

Among the farmers that never took their animals for vaccination in Murang’a, disinterest (11 FGDs;6-M, 5-W), qualified as farmers being fully aware that a livestock vaccination exercise was taking place and being able to manage the transactional costs associated with the animal vaccination but still choosing not to have them vaccinated anyway, was reported. This attitude came from farmers not seeing any visible difference between animals that were vaccinated and those that were not, therefore, concluding that livestock vaccination was unnecessary. A similar observation was made in Kwale in 12 FGDs (6-M, 6-W) in areas where vaccine provision was irregular.

(f) Lack of or limited access to vaccination information:

Most farmers in Murang’a received information on impending vaccination exercises as demonstrated by the low number of FGDs (2 FGDs; 1-M, 1-W) that identified lack of vaccination information as a barrier. From the sub-County veterinary offices, vaccination information was relayed through veterinary personnel to the public administration (chiefs, assistant-chiefs, and village elders), schools, churches, mosques, posters pinned in public spaces, and from farmer to farmer. The information comprised of the vaccine to be offered, the species targeted, cost of vaccine, date and place of vaccination. However, men farmers reported in one FGD that the time when one got the information was important because it affected the ability to raise the money required for the vaccination exercise. Longer notices were preferred to shorter ones. In Kwale, farmers from 14 FGDs (8-M, 6-W) reported that lack of vaccination campaigns information contributed to low uptake of vaccines. Veterinary officers relayed vaccination information to public administrators for onward dissemination to community members, but the administrators were not efficient in reaching out to community members. Further, farmer to farmer sharing of information on vaccination was not common.

In Ibanda, six FGDs (3-M, 3-W) and in Arua, five FGDs (2-M, 3-W) reported that delayed, limited, or lack of access to vaccination campaign information affected farmers ability to prepare for vaccine uptake. In both areas, farmers received vaccination campaign information from the Local Council (LC) Chairmen 1 and 2. The leaders shared the information through churches, mosques, and social events like weddings and funerals. Information sharing was also achieved through farmer to farmer communication. Notices were also placed in marketplaces where people tended to congregate. In consultation with Resident District Commissioners (RDC), veterinary officers could be allocated air-time on radio to make an announcement of upcoming vaccination campaigns. The method was, however, found not to be effective because not many community members listened to the local government radio in the hours that the announcements were made.

(g) Mistrust of veterinary vaccines and veterinary officers:

In Kwale, eight FGDs (5-M, 3-W), comprising of more men’s groups than women’s, identified mistrust of veterinary vaccines as a barrier to uptake. The mistrust was fueled by the belief that the veterinary department offered farmers vaccines for free because they were being tested for efficacy, about to expire, faulty, or were intended to reduce the livestock population in the area by inducing infertility and death. More men than women farmers were suspicious of the vaccines because they mainly targeted cattle, a major asset whose ownership and control was culturally ascribed to them. The same barrier was reported in eight FGDs (4-M, 4-W) in Ibanda. The farmers mistrusted vaccines provided by the veterinary personnel, particularly those in private practice because they were not effective when administered to the animals, a factor blamed on poor storage and maintenance of the cold chain. In Ibanda, the mistrust of vaccines was closely linked to mistrust of veterinary personnel, a barrier identified in five FGDs (3-M, 2-W). The mistrust was cultivated by previous farmer experience with vaccines offered by some veterinary personnel not being effective and some rogue veterinary personnel offering farmers placebos instead of the needed medicines and vaccines. For instance, farmers reported that rogue veterinary personnel provided them (farmers) with water for clear colored vaccines and a yellow colored soft drink for injectable tetracycline.

(h) Livestock ownership patterns:

In Ibanda, due to existing culturally defined livestock ownership patterns, women could access livestock and livestock products but had limited decision making capacity over cattle (5 FGDs; 1-M, 4-W). Therefore, even when women wanted the families’ cattle vaccinated, if the men (heads of household) did not consent, their animals did not benefit from the vaccination exercise. This barrier affected women farmers more because of power relations between them and their spouses at a household level.

(i) Poor access roads:

Farmers in Kwale (7 FGDs; 4-M, 3-W) and Arua (2 FGDs; 1-M, 1-W), reported that in the planting season, there was the challenge of a lack of defined paths to move livestock through since most land was unfenced and occupied by crops at the time. Livestock was, therefore, likely to destroy or feed on the planted materials leading to conflicts among community members.

(j) Cultural beliefs:

In Murang’a, farmers from nine FGDs (4-M, 5-W) reported that bringing their healthy and productive animals to public spaces exposed them to envy from other farmers resulting in them “being looked at with a bad eye”, locally referred to as “githemengu”, which was believed to lower milk production and even lead to death of the animals. It would also expose animals to injuries and theft, therefore, compromising the animals’ safety and security (4 FGDs; 3-M, 1-W). In Ibanda, two FGDs (1-M, 1-W), reported that in observation of cultural beliefs, specific animals in some households could not be vaccinated so as not to compromise the totem status they held. In Kwale, farmers feared exposing all their livestock for fear that persons with ill intent would use witchcraft to reduce the wealth of the owner (2 FGDs; 1-M, 1-W).

(k) Religious beliefs:

In Murang’a, members of the “Akorino” religious sect, who did not believe in utilizing medical services for humans and veterinary services for animals, were reported not to avail their animals for vaccination in nine FGDs (5-M, 4-W).

(l) Waiting time in vaccination points:

In Murang’a (4 FGDs; W) and Kwale (3 FGDs; W), women farmers decried the time spent in the vaccination centers waiting for the service to be delivered because it compromised their ability to engage in other domestic responsibilities.

(m) Inadequate vaccines:

In Arua, Kwale, and Murang’a, during vaccination campaigns, farmers in six FGDs (4-M, 2-W), two FGDs (1-M, 1-W) and one (FGD; W), respectively, reported that the vaccine available was not always adequate to cover all eligible animals hence some farmers missed out. Women in one FGD in Ibanda also reported that vaccines that could be bought in veterinary supplies shops were not always available because the proprietors preferred to only sell fast moving items. This caused a delay in vaccine access and delivery as reported in five men’s FGDs in Ibanda. A similar challenge was reported in a men’s FGD in Kwale where due to delays in vaccine procurement processes, the veterinary department sometimes acquired vaccines after the disease that was intended for control had spread extensively.

(n) Farmer availability:

In Murang’a, Kwale, and Ibanda farmers from four FGDs (2-M, 2-W); one (FGD; M), and one (FGD; W), respectively, stated that in some instances, vaccination dates were set on days when some farmers had commitments that made it impossible to participate in the vaccination exercise. Thus, farmer availability determined vaccine uptake. The unavailability for vaccine uptake could also be triggered by weather patterns. For instance, in Kwale, during the dry season, farmers practicing pastoralism moved their animals in search of water (1 FGD; M). Thus, if vaccine delivery was conducted far from where they had moved the animals to, the probability of not presenting the animals for vaccination was high.

(o) Difficulties in restraining animals:

In Arua (3 FGDs; 1-M, 2-W) and Kwale (1 FGD; W), farmers reported difficulties in manually restraining animals for vaccination, particularly in places where there were no crushes. This challenge affected women farmers more than men farmers since it was an energy intensive exercise.

(p) Shame of having unhealthy animals:

In Murang’a from two FGDs (1-M, 1-W), farmers with less appealing animals as a result of poor management reportedly did not take their animals for vaccination for fear of becoming objects of shame in the community.

(q) Lack of knowledge of vaccine importance:

In Arua (11 FGDs; 5-M, 6-W), Ibanda (2 FGDs; W), and Kwale (1 FGD; W), lack of knowledge of the importance of vaccines was identified as a deterrent to vaccine uptake. This was attributed to the lack of awareness-creation sessions with the farmers.

(r) Inadequate numbers of veterinary officers:

In Arua and Kwale, lack of or having few well-trained veterinary officers was identified as a barrier to vaccine uptake because of difficulties in accessing services as reported in eight FGDs (4-M, 4-W) and five FGDs; (3-M, 2-W), respectively.

(s) Lethargy:

Lethargy, qualified as being in the knowledge that there was an ongoing livestock vaccination campaign but not participating because of not wanting to tire one-self (4 FGDs; 3-M, 1-W) was also a deterrent to vaccine uptake in Arua.

(t) Fear of animal loss:

In Kwale, farmers (6 FGDs; 3-M, 3-W) identified fear of animal losses in vaccination sites and while moving to and from vaccination points as a barrier.

(u) Shortness of vaccination campaign duration:

Farmers in Kwale decried the shortness of the time allocated for vaccination campaigns (5 FGDs; 3-M; 2-W) because they felt it was not adequate to have all their animals vaccinated and would like more time allocated for the service.

(v) Use of herbal medicines:

In one FGD for men farmers, the use of herbal medicines for livestock treatment was identified as a deterrent for livestock vaccines uptake. The perception was driven by the belief that herbal medicines were capable of treating and preventing livestock diseases, hence there was no need for using vaccines.

(w) Preference for curative rather than preventive treatment:

Women farmers in two FGDs, one in Murang’a and one in Arua, reported having a preference for the uptake of curative rather than preventive services since it was not always the case that when animals were vaccinated that there would be an outbreak of the disease vaccinated against.

### 3.3. Barrier Ranking by Men and Women Farmers Based on Self-Assessment and Triangulation Using Pairwise Ranking Exercises

Of the identified barriers in Murang’a, men farmers reported the cost of vaccine as their main barrier to livestock vaccine uptake. However, women farmers thought that the main barrier to men’s vaccine uptake was their (men’s) unavailability, implying that women may be the ones taking animals for vaccination in the absence of their spouses. Both men and women farmers identified women’s key barrier to vaccine uptake as the choice of place of vaccination due to difficulties in moving animals to vaccination points. From pairwise ranking exercises conducted by men farmers, the barrier with the highest ranking at community level was choice of livestock vaccination points, which ranked first in 3/7 FGDs and second in 2/7 FGDs (Table 3). Closely following this was cost of vaccine, which was ranked first in 2/7 FGDs, second in 1/7 FGDs and third in 2/7 FGDs. Among women farmers, the highest ranked barrier was the choice of vaccination points, which was ranked first in 4/7 FGDs, second in 1/7 FGDs and third in 2/7 FGDs. The pairwise ranking results show agreement on the choice of vaccination place, making it the most pressing vaccine uptake barrier for the men and women farmers, closely followed by cost.

In Kwale, the key vaccine uptake barrier for men farmers, as identified by both men and women farmers, was lack of vaccination crushes. For women, the key barriers, which held similar weight, were lack of vaccination crushes and limited access to vaccination campaigns information. From pairwise ranking exercises, the barriers with the highest ranking for the community as identified by men discussants were: lack of vaccination crushes and livestock vaccination campaign information, which were ranked similarly; first in 1/7 FGDs, second in 2/7 and third in 2/7 FGDs. The barrier with the highest pairwise rank for women discussants was a lack of vaccination campaign information, which was ranked first in 4/7 FGDs and was closely followed by a lack of livestock vaccination crushes, which was ranked second in 5/7 FGDs and third in 1/7 FGDs. The pairwise ranking outcomes were, thus, in agreement with the self-assessment done by men and women farmers.

In Ibanda and Arua, the key vaccine uptake barrier for men as identified by both men and women farmers was cost of vaccine. For women farmers, the key barrier to vaccine uptake as identified by men farmers was the limited decision-making capacities women have in cattle production due to differences in livestock ownership patterns, where culturally cattle are owned by men. In addition to having limited decision-making capacities, women identified inhibitive costs of vaccine as a key barrier they were faced with. From pairwise ranking exercises conducted in Ibanda, the barrier with consistent high ranking as identified by men discussants was cost of vaccine; which was ranked first in 2/7 and second in 3/7 FGDs. Among women discussants, cost also emerged as the greatest barrier; ranked first in 2/7 FGDs and second in 4/7 FGDs. This confirms vaccine cost as the greatest barrier for vaccine uptake for men and women farmers, as they had reported based on self-assessment. Although from self-assessment women also identified limited capacity in decision making over livestock as a barrier, it was ranked first, second, and third in only 1/7 FGDs each, respectively. This ranking was lower than for having limited access to vaccination information, which was ranked first in 3/7 FGDs. From pairwise ranking exercises in Arua, cost of vaccines was consistently ranked highly in all seven men FGDs; first in 3/7 FGDs, second in 2/7 FGDs and third in 2/7 FGDs. In women groups, it was ranked highly five times; first in 2/7 FGDs, second in 1/7 FGDs and third in 2/7 FGDs. Combined, it ranked first in 5/14 FGDs, second in 3/14 FGDs and third in 4/14 FGDs times. This finding thus confirms that indeed, cost was the biggest vaccine uptake inhibitor for men and women farmers in Arua as identified by the farmers themselves.

## 4. Discussion

The four case studies (Murang’a, Kwale, Ibanda, and Arua), demonstrate that geographically there were contextual livestock uptake barriers in each site. This indicates that for small holder farmers, mass provision of vaccines by the national and local veterinary departments does not guarantee uptake. Of the four case studies, farmers in Murang’a demonstrated more in-depth knowledge of barriers to vaccine uptake. This can be linked to their practice of keeping cross-bred dairy animals in intensive systems for which vaccines played a major role in health and productivity enhancement.

There were both differences and similarities in the identification of barriers faced by men and women farmers. Men’s discussion groups in Murang’a, Arua, and Ibanda identified more barriers than women farmers although the difference was not big. This can be attributed to the gendered division of labor in control and management of disease in large stock, which is primarily considered men’s responsibility. In Kwale, the same division of labor pattern applied but due to the practice of pastoralism in some of the areas, women were sometimes left with part of the livestock in the homestead as men moved other animals in search of better pasture and water sources, and thus faced greater challenge in the management and control of livestock diseases in the absence of the men. This may explain the high number of livestock vaccine barriers identified by women farmers in Kwale compared to men farmers.

Among the identified barriers to vaccine uptake, costs emerged as a challenge in each case study as well as the greatest barrier for Arua, Ibanda, and Murang’a farmers. Cost becomes a critical determinant of vaccine uptake since in households where livestock vaccination costs are higher than available disposable income, farmers may forfeit vaccination or only have part of their animals vaccinated [31,32,33,34]. A study on the uptake of the Contagious Bovine Pleuropneumonia (CBPP) vaccine in Kenya demonstrated that wealth levels in terms of herd sizes and access to alternative income sources were an important determinant of farmer ability to adopt the technology [31]. While both men and women farmers raised the concern of costs, more men than women farmers identified it as a concern since they (men farmers) predominantly bore costs associated with livestock disease management. This is, however, not always the case as in a study on the use of the Infection and Treatment Method (ITM) to vaccinate animals against East Coast Fever (ECF) in Kenya, women identified cost as their main barrier to adoption [35].

Community perceptions of how national and local government services should be delivered to them can shape attitudes towards the levied costs. In Murang’a, Ibanda, and Arua, farmers held the belief that services delivered by the national or local governments should be fully subsidized and farmers should not have to bear any financial costs. Consequently, farmers may have perceived vaccination costs to be high not because they did not have the ability to pay for the service but because they believed they should not have to pay. However, the example from Kwale in which community members received with suspicion fully subsidized livestock vaccines demonstrated that in some contexts, it can be counter-productive not to charge a minimal cost for vaccines delivered.

In Murang’a, Kwale, and Arua, farmers had to contend with covering some distances with their animals to vaccination points. While the distances were relatively short in Murang’a, women farmers reported difficulties in taking zero grazed dairy animals for vaccination since the cattle were not used to walking any, let alone, long distance. Hence, both men and women farmers preferred that livestock vaccines are provided at home even if the cost of service would be higher. In Kwale and Arua, farmers kept indigenous cattle breeds (zebu) that were accustomed to frequent movement. However, due to lengthy distances to vaccination points, farmers kept away. This finding resonated with Waithanji et al. [31] who reported that farmers may avoid having their animals vaccinated if set vaccination schedules interfered with herding patterns; would expose the animals to raid risk, and if the animals had poor body condition. For farmers in Murang’a, Ibanda, and Kwale, a concern related to animals from different households congregating was the possibility of disease spread through animal contact and using a single needle to vaccinate many animals whose health status was unknown. A study on Highly Pathogenic Avian Influenza (HPAI) has demonstrated that disease spread is possible through the two infection routes [36] and the same is possible with RVF.

In three case studies, farmers identified vaccine side effects as barriers to vaccine uptake. Abortions of in-calf animals were identified in Murang’a, Kwale, and Ibanda case studies. Decline in milk production; body swelling at vaccination sites; and animals becoming sick and dying were identified in Murang’a and Ibanda; while tail tip loss was identified in Kwale and Ibanda. Of the identified side effects, the Smithburn vaccine has been reported to cause abortions and being a live attenuated vaccine, if administered to an animal that is already sick with RVF, can cause severe sickness and death [5]. This indicates a need to explain to farmers the possible side effects before vaccination as none or partial disclosure may have a negative influence on farmer attitudes towards vaccines in future. Perceptions of the possible effects of a vaccine can affect its acceptability [31,37]. If a product causes disease, it introduces product mistrust and can lead to a higher rejection rate compared to the harm caused [31]. Such experiences can also become sources of negative publicity against a specific vaccine and vaccines in general at the community level. A study on HPAI reports that for vaccination against disease to be successful, farmers must be provided with vital information, which constitutes of the duration it takes for an animal to gain protective immunity; the disease that the vaccine confers protection against; the fact that a healthy looking animal can be incubating disease; and that all animals do not respond the same to vaccines and some may react [36].

In Murang’a and Kwale, farmers reported that disinterest in livestock vaccines contributed to low uptake. This was largely due to having limited knowledge of the importance of vaccines as reported in Arua. A study on the impacts of adopting best health and husbandry practices among cattle farmers in Cambodia compared farmers who were provided with livestock vaccines only, without raising knowledge levels on their importance, and farmers who were provided with information and vaccines and found that uptake was higher in the latter group [38]. Further, commercial farmers are more likely to be knowledgeable of vaccines and their benefits compared to subsistence farmers [33]. This demonstrates the need for raising farmer awareness prior to providing them with vaccines. A strategy would be to provide farmers with detailed information concerning the disease being vaccinated against instead of informing them only of the name of the disease, species being vaccinated, date, place, and time of vaccination as provided in a vaccination campaign announcement. Another potential cause of disinterest could be the timing of vaccine delivery [31]. In the absence of disease, some farmers may not take having their animals vaccinated as seriously as when cases of the disease have been reported due to a lowered perception of risk. Farmers are also likely to adopt vaccines if they feel that they are getting value for the money invested. For example, a study in Tanzania and North Eastern Kenya demonstrated that part of farmer motivation to vaccinate their animals against ECF was ear tagging to indicate that an animal was vaccinated, a factor that caused the animals to fetch 50% more than unvaccinated ones during sale [32].

Lack of or limited access to vaccination campaign information was also identified as a challenge in all the case studies and the main barrier to vaccine uptake for women in Kwale. A similar finding on a study on the use of the Infection and Treatment Method for East Coast Fever also established that a lack of awareness of the availability of the technology constrained adoption [35]. Vaccination information is mainly disseminated orally by local administrators and announcements in schools and churches/mosques and community activities. The short coming with these techniques is that they may have a narrow reach if the persons that require the information are not at the dissemination points when announcements are being made. The use of posters/written announcements in public spaces was also limited in that only literate persons can get information in that way. For effective planning of dissemination of vaccination information, organizers should be cognizant of the fact that men and women have differential access to information based on their roles and responsibilities [31]. For example, a study among poultry producers in Tanzania reported while men poultry producers had heard about the availability of the New Castle vaccine locally, women farmers had not [39]. This demonstrated that it is possible for women not to have critical information related to the livestock they manage largely due to their lower literacy levels compared to men [39]. The study observed that in all the study sites, use of mobile phones to share vaccination information was very limited despite wide ownership at community level thus indicating a potential strategy for improvement of dissemination.

In Kwale, Ibanda, and Arua, the question of trust of vaccines and veterinary personnel emerged as a barrier to vaccine uptake. In Kwale, the mistrust of vaccines was driven by local beliefs held predominantly by men farmers that fully subsidized vaccines are harmful to their animals. In Ibanda, it was because of repeated experiences of vaccine and medicine inefficiency attributed to poor cold chain maintenance and rogue veterinary personnel allegedly offering farmers placebos. The case in Kwale demonstrated a need for increasing farmer knowledge on the importance and usefulness of vaccines to counter negative beliefs. In Ibanda, it is likely that the alleged placebos were genuine vaccines and medicines that had been rendered ineffective due to poor cold chain maintenance hence the veterinary officers may not be rogue as alleged but rather faced with infrastructure and cold chain maintenance equipment challenges. However, this does not rule out the possibility of having rogue animal health service providers in the locality. Provision of ineffective vaccines is a loss to farmers as they do not get value for money invested in disease prevention and it can also lead to a surge of avoidable livestock disease incidences and deaths [40].

Another barrier that can lead to mistrust of veterinary departments is having a limited number of veterinary personnel that are unable to efficiently meet farmers’ needs as reported in Kwale and Arua. Having limited contact time with farmers may affect the level of importance/priority accorded to the information veterinary officers/departments provide to communities since they are considered strangers. Having limited access to veterinary services is a major constraint to the uptake of veterinary technologies [31]. A study on the uptake of vaccines in India records that if farmers are located far from agricultural extension services, they are less likely to adopt improved livestock production technologies such as vaccines [37,41].

The unique case in Ibanda where the greatest barrier to vaccine uptake for women was having limited decision-making capacity over cattle due to the prevailing livestock ownership patterns indicates that intra-household relations have a role to play in determining vaccine uptake, particularly in male headed households but may not be a limitation for women in all cases. Individuals that make decisions on what to produce and how to utilize the resultant products tend to decide on the agricultural technologies to adopt at a household level [42]. However, it is not always the case that having limited decision-making capacity over livestock is negative for women. It could also be a deliberate and strategic choice on their part. For example, in instances where women feel they are not benefiting from livestock or livestock products such as getting milk for domestic use and access to milk income or are in a precarious marital situation, they may deliberately keep away from investing in livestock technologies such as vaccines even when they have the ability to utilize them [31].

Triangulating the self-reported barriers through pair-wise ranking demonstrated that men and women farmers were acutely aware of the barriers that inhibited their vaccine uptake. This showed the importance of involving them at community level in identifying the challenges they face in order to develop interventions/services that are contextually appropriate for them. Designing interventions with a poor understanding of the context can lead to poor uptake and may unintentionally or unknowingly reinforce gender inequalities.

### Implications of the Study Findings on Uptake of the RVF Vaccine

(a) Addressing inequalities in vaccine access:

The study has demonstrated that men and women farmers experience barriers in accessing vaccines provided by veterinary departments. Some barriers affect both men and women farmers and others affect one gender group only based on prevailing gender norms and division of labor. An example of those affecting both men and women farmers was cost in Arua. Among the gender specific instances, women in Kwale had more difficulties than men in accessing vaccination campaign information while women in Ibanda had limited decision making capacity over livestock disease management and control due to prevailing livestock ownership patterns. Men, on the other hand, were affected more by cost of vaccines than women because the role of livestock disease management and control was culturally ascribed to them. Thus, it is important for veterinary departments to understand the context within which farmers access livestock vaccines to enhance active participation by men and women based on their roles and responsibilities without reinforcing existing gender inequalities. In contexts where men and women are faced with a similar primary barrier such as cost in Arua and the lack of vaccination crushes in Kwale, it should not be assumed that resolving it will translate to equal vaccine uptake as the primary barrier may be masking other gender specific barriers whose impact becomes more prominent in its absence.

(b) Enhancing RVF vaccine provision:

Without government approval of the use of an RVF vaccine and listing RVF as part of those diseases the government is responsible for in Uganda, farmers will remain at risk since there will be no reliable means of prevention. Although in Kenya there is an approved vaccine for use, if it is not provided to farmers in a timely manner and in adequate quantities to cover the animals at risk, they (farmers) will also remain at risk of both livestock and human RVF disease occurrences.

(c) Increasing farmer awareness of RVF risk factors:

Since not all men and women farmers avail their livestock for vaccination, there is a need to increase their knowledge on RVF signs and symptoms, susceptible species, risk factors, and disease prevention and control practices, which include vaccine uptake. Veterinary departments can take advantage of livestock vaccination mobilization exercises to provide farmers with information on how livestock vaccination confers protection from RVF disease in both humans and livestock and practices that put farmers at risk of infection. This will supplement knowledge transfer in areas where extension services are minimal or non-existent, enhance participatory disease surveillance, and the ability of farmers to reduce exposure to disease during outbreaks.

(d) Farmer preference for vaccinating one of the susceptible species:

The case studies have shown that men and women farmers in Kenya and Uganda tended to present cattle for vaccination rather than goats and sheep. This implies that during a vaccination campaign, particularly in cases where farmers are required to pay a fee, two of the susceptible species found in the study sites can be left out hence compromising the herd immunity created through cattle vaccination.

(e) Farming systems:

In Kwale, where some men and women farmers practice pastoralism and periodically move livestock to Tanzania and back, there is a need for a coordinated response between veterinary departments along the border in Kenya and Tanzania in the control of trans-boundary diseases, RVF included.

(f) RVF vaccine side effects:

Since men and women farmers identified abortions as an undesirable side effect of vaccines, it is important that if using the Smithburn vaccine, this information be communicated to farmers to avoid negative publicity in future. This, however, needs to be communicated in a non-alarming way that emphasizes that the benefits of the vaccine outweigh the losses incurred from any abortion that may occur after vaccination.

(g) Implementing vaccination campaigns in ways that are accessible to the majority of farmers:

The study demonstrated the importance of ensuring that vaccination campaign information reaches both men and women farmers and that the service is offered in areas that are easily accessible to farmers at a cost that is affordable to all households regardless of their wealth status. The information should be packaged in a way that is easily comprehensible even for farmers with low education levels.

## 5. Conclusions

The study has demonstrated that availability of vaccines does not guarantee uptake at community level due to social, spatial, economic, and vaccine safety and efficacy barriers faced by men and women farmers. Vaccine uptake is a complex responsibility shared by men and women farmers, veterinary departments, county/district and national governments, and vaccine producers. At a national level, setting up requisite guidelines on the type of vaccine to use for RVF prevention and regulations on its provision and use can influence farmer access to the vaccine. At district/county level, how veterinary departments organize vaccine delivery campaigns in terms of information dissemination, choice of vaccination points, costs of vaccines, and pre-existing relationships with community members can also determine uptake. At a community and household level, the process is influenced by intra-household relations and decision-making capacities between men and women, level of knowledge on the importance of vaccines, financial ability and willingness to pay for vaccination services, ease of accessing vaccination points, perceived vaccine side effects, and trust in vaccines and veterinary personnel, all of which can affect men and women farmers similarly or differentially depending on context. If Kenya and Uganda are to have effective vaccination campaigns against RVF, these barriers should be considered in the design and delivery process.

## 6. Limitations

This study applied a cross sectional study design, which informed about the farmer experience with vaccine uptake at a time. The researchers did not follow farmers during an active vaccination campaign period to interview them about the barriers they were facing at the time. All these are based on recalled experience. However, the information collected is useful because it enables us to understand how the structure of vaccine delivery and the social, cultural, and economic environments farmers find themselves in affect vaccine uptake.

## Figures and Tables

**Table 1:** Demographic Characteristics.

Demographic Characteristics				Kenya		Uganda	
				Murang’a	Kwale	Ibanda	Arua
1	Number of Focus Group Discussions		Men	7	8	7	7
			Women	7	8	7	7
			Total	14	16	14	14
2	Number of Focus Group Discussion participants		Men	76	81	84	81
			Women	78	82	83	80
			Total	154	163	167	161
3	Participants’ education levels	Primary	Men	44.4%	54.3%	71.4%	54.3%
			Women	61.5%	48.8%	42.2%	65%
			Combined	53.3% (*N* = 150) *	51.5% (*N* = 163)	59.6% (*N* = 167)	59.6% (*N* = 161)
		Secondary	Men	48.6%	28.4%	19%	32.1%
			Women	34.6%	18.3%	22.9%	18.8%
			Combined	41.3% (*N* = 150) *	23.3% (*N* = 163)	21% (*N* = 167)	25.5% (*N* = 161)
		Tertiary	Men	5.6%	3.7%	3.6%	9.9%
			Women	0	6.1%	3.6%	3.8%
			Combined	2.7% (*N* = 150) *	4.9% (*N* = 163)	3.6% (*N* = 167)	6.8% (*N* = 161)
		Adult education	Men	1.4%	2.5%	0	0
			Women	0	0	1.2%	0
			Combined	0.7% (*N* = 150) *	1.2% (*N* = 163)	0.6% (*N* = 167)	0 (*N* = 161)
		None	Men	0	11.1%	6%	3.7%
			Women	3.8%	26.8%	30.1%	12.5%
			Combined	2% (*N* = 150) *	19% (*N* = 163)	18% (*N* = 167)	8.1% (*N* = 161)
5	Participants’ age Ranges (in years)		Men	26–79	20–73	20–82	21–68
			Women	23–78	20–78	22–76	20–65
			Combined	23–79	20–78	20–82	20–68
6	Participants’ age (in years)	Mean	Men	53.8	47	48.2	39.7
			Women	46.8	45.4	46	36.7
			Combined	50.4	46.2	47.1	38.2
		Median	Men	53.5	45	46	40
			Women	47	45.5	46	35
			Combined	50.1	45	46	38
7	Types of susceptible livestock kept in order of proportions			Cattle Goats Sheep	Cattle Goats Sheep	Cattle Goats Sheep	Cattle Sheep Goats
8	Livestock species preferred for vaccination			Dairy cattle	Cattle Goats	Cattle	Cattle Goats

* Four participants preferred not to disclose their ages and education status.

**Table 2:** List of livestock vaccine uptake barriers identified by men and women farmers.

Barriers to Livestock Vaccine Uptake		Kenya				Uganda				Total Number of FGDs Identifying Barrier		
		Murang’a		Kwale		Ibanda		Arua				
		Men FGDs	Women FGDs	Men FGDs	Women FGDs	Men FGDs	Women FGDs	Men FGDs	Women FGDs	Men FGDs	Women FGDs	All FGDs
1	Cost of vaccines	7	7	-	-	7	7	7	6	21	20	41
2	Lack of or limited access to vaccination information	1	1	8	6	3	3	2	3	14	13	27
3	Vaccine side effects	6	5	1	2	5	6	-	-	12	13	25
4	Disinterest in livestock vaccines	6	5	6	6	-	-	-	-	12	11	23
5	Long distances to vaccination points	-	-	8	8	-	-	3	3	11	11	22
6	Fear of disease spread through animal contact	7	4	4	5	-	-	-	-	11	9	20
7	Fear of disease spread through sharing one needle for many animals	7	6	-	-	2	1	-	-	9	7	16
8	Mistrust of vaccines	-	-	5	3	4	4	-	-	9	7	16
9	Lack of awareness of the importance of vaccines	-	-	-	1	2	-	6	6	8	7	15
10	Choice of place of livestock vaccination	7	7	-	-	-	-	-	-	7	7	14
11	Lack of vaccination crush(es)/dips	-	-	7	7	-	-	-	-	7	7	14
12	Lack of/limited number of veterinary officers	-	-	3	2	-	-	4	4	7	6	13
13	Bad eye belief (Githemengu)	4	5	-	-	-	-	-	-	4	5	9
14	Religious belief (Akorino)	5	4	-	-	-	-	-	-	5	4	9
15	Lack of proper paths to move livestock in the cropping season	-	-	4	3	-	-	1	1	5	4	9
16	Limited/Inadequate vaccines quantities	-	1	1	1	-	-	4	2	5	4	9
17	Dip use charges	-	-	3	4	-	-	-	-	3	4	7
18	Waiting time at vaccination points	1	3	-	3	-	-	-	-	1	6	7
19	Fear of animal loss	-	-	3	3	-	-	-	-	3	3	6
20	Late/Delayed provision of vaccines after disease spread is extensive	-	-	1	-	5	-	-	-	6	-	6
21	Short vaccination campaign time	-	-	3	2	-	-	-	-	3	2	5
22	Conflicts resulting from livestock ownership patterns	-	-	-	-	1	4	-	-	1	4	5
23	Mistrust of veterinary personnel	-	-	-	-	3	2	-	-	3	2	5
24	Farmer unavailability	2	2	-	-	-	1	-	-	2	3	5
25	Safety and security of the animal	3	1	-	-	-	-	-	-	3	1	4
26	Lethargy in farmers	-	-	-	-	-	-	3	1	3	1	4
27	Difficulties in restraining animals	-	-	-	1	-	-	1	2	1	3	4
28	Shame of having unhealthy animals	1	1	-	-	-	-	-	-	1	1	2
29	Fear of animals being bewitched	-	-	1	1	-	-	-	-	1	1	2
30	Totem animals cultural belief	-	-	-	-	1	1	-	-	1	1	2
31	Use of herbal medicines to treat animals	1	-	-	-	-	-	-	-	-	-	1
32	Lack of someone to take the animals to vaccination points	-	-	1	-	-	-	-	-	1	-	1
33	Water shortage in the dry season	-	-	1	-	-	-	-	-	1	1	1
34	Irregular provision of vaccines	-	-	-	1	-	-	-	-	-	1	1
35	Preference for curative to preventive veterinary services	-	1	-	-	-	-		1	-	2	1
36	Timing of receipt of vaccination information	1	-	-	-	-	-	-	-	1	-	1
37	Vaccine unavailability	-	-	-	-	-	1	-	-	1	1	1

**Table 3:** A comparison between self-assessment of barriers to livestock vaccine uptake and pairwise ranking exercise outcomes.

Site		Gender	Barrier Ranking by Self-Assessment in Focus Group Discussions (FGDs)	Barrier Ranking from Pairwise Ranking Exercises	Ranks by FGDs		
					1st	2nd	3rd
1	Murang’a	Men	Cost of vaccine	Choice of vaccination place (1st)	3	2	-
				Cost of vaccine (2nd)	2	1	2
		Women	Choice of livestock vaccination place	Choice of vaccination place (1st)	4	1	2
2	Kwale	Men	Lack of vaccination crushes	Lack of livestock vaccination crushes (1st)	1	2	2
				Lack of vaccination information (1st)	1	2	2
		Women	A tie between lack of vaccination crushes and lack of vaccination information	Lack of livestock vaccination information (1st)	4	-	-
				Lack of livestock vaccination crushes (2nd)	-	5	1
3	Ibanda	Men	Cost of vaccine	Cost of vaccine (1st)	2	3	-
		Women	Having limited decision making capacity over cattle	Cost of vaccine (1st)	2	4	-
				Having limited access to vaccination information (2nd)	3	-	-
				Having limited decision making capacity over cattle (3rd)	1	1	1
4	Arua	Men	Cost of vaccine	Cost of vaccine (1st)	3	2	2
		Women	Cost of vaccine	Cost of vaccine (1st)	2	1	2

## Supplementary Materials

The following are available online at https://www.mdpi.com/2076-393X/7/3/86/s1, Table S1: Murang’a pairwise ranking data, Table S2: Kwale pairwise ranking data, Table S3: Ibanda pairwise ranking data, Table S4: Arua pairwise ranking data
